# Disease activity and treatment in patients with juvenile idiopathic arthritis before transfer to adult care: the first survey in China

**DOI:** 10.3389/fped.2025.1535223

**Published:** 2025-04-03

**Authors:** Xiya Luo, Xiwen Luo, Qiang Luo, Xuemei Tang

**Affiliations:** ^1^ Department of Rheumatology and Immunology, Children’s Hospital of Chongqing Medical University, Chongqing, China; ^2^ National Clinical Research Center for Child Health and Disorders, Children’s Hospital of Chongqing Medical University, Chongqing, China; ^3^ Ministry of Education Key Laboratory of Child Development and Disorders, Children’s Hospital of Chongqing Medical University, Chongqing, China; ^4^ Chongqing Key Laboratory of Child Rare Diseases in Infection and Immunity, Children’s Hospital of Chongqing Medical University, Chongqing, China

**Keywords:** juvenile idiopathic arthritis, pre-transfer, disease activity, treatment, biologics

## Abstract

**Objectives:**

To analyze disease activity and treatment in patients with juvenile idiopathic arthritis (JIA) before transfer to adult care.

**Methods:**

We retrospectively collected the clinical data of 230 JIA patients (range 14–18 years) in our center from January 2013 to December 2022. We evaluated the clinical features, disease activity, and medication use across various JIA subtypes.

**Results:**

230 patients with JIA were included, and 144 (63%) were male. The distribution of JIA subtypes was dominated by enthesitis-related arthritis (32%), polyarthritis (31%), systemic JIA (27%), and oligoarthritis (10%). Disease activity assessment showed that 87 JIA (38%) were in active disease; while 143 JIA (62%) were in inactive disease, of which 59 patients achieved clinical remission on medicine and 13 patients achieved clinical remission off medicine. Conventional synthetic disease-modifying anti-rheumatic drugs were used in 83% of JIA patients, and biologics in 56%. Clinical characteristics and medication use differed between different subtypes of JIA. The oligoarthritis group had earlier disease onset (*P* = 0.020) and longer disease duration (*P* = 0.009) compared to other subtypes. Patients in the RF-positive polyarthritis group had a significantly lower rate of disease inactivity (39%, *P* = 0.004) than the other subtypes, and a relatively lower proportion of patients achieved clinical remission on medication or discontinuation of medication (18%, *P* = 0.024).

**Conclusions:**

Some JIA patients were still in active disease before transfer to adult clinics, failing to achieve clinical remission and discontinuation of medication, and required continued treatment. Patients in the RF-positive polyarthritis group were less likely to achieve clinical remission.

## 1 Introduction

Juvenile idiopathic arthritis (JIA) is one of the common rheumatic diseases in children. With advances in treatment, the prognosis of patients with JIA has improved. However, some patients remain in active disease status in adulthood and fail to achieve clinical remission off medication, which needs to be referred to adult rheumatology for continued treatment (1–5).

Transition is defined as “the purposeful, planned movement of adolescents and young adults with chronic physical and medical conditions from child-centered to adult-oriented health-care systems” (6). There is currently no established consensus regarding the optimal timing for this phase. In different studies, the timing of transition varies from 14 to 20 years of age (7, 8). Effective transitional care improves the quality of life of patients with JIA, whereas transition loss may result in negative outcomes (9, 10).

However, there is a lack of awareness of transition among patients, their families, and even rheumatologists in the current healthcare situation. Studies have shown that only 16% of patients have considered transition-related issues before transitioning to adulthood (11). About half of the patients and their families have little or no knowledge of the concepts of transition (12). Even up to 44% of adult rheumatologists were hesitant or anxious during providing care to JIA patients, and they expressed a lack of readiness and related experience in accepting adult patients with JIA (13). Transition services are also not yet robust globally. Data from the Utah Children's Health Survey (2018–2019) found that only 11.5% of patients aged 12–17 years with childhood-onset rheumatic diseases, received the services to facilitate their transition to adult healthcare (14). In China, most patients, their families, and even some rheumatologists do not know much about transitional care. Moreover, the healthcare system for pediatric rheumatology is often separate from adult healthcare facilities in China. Patients of JIA must be referred to adapt to the new healthcare environment as adults, making the transition more difficult.

According to the transition recommendations from the American Academy of Pediatrics (15), the transition process should include three stages: preparation, transfer, and integration into adult health care. Therefore, to enhance rheumatologists' awareness of the transition of JIA and to provide a reference for adult rheumatologists in the diagnosis and treatment of JIA patients, our study evaluated the disease activity and treatment at the last pediatric visit in patients with JIA (age range 14–18 years). This was the first study on JIA patients before transfer to adult care in China.

## 2 Methods

### 2.1 Study population

We retrospectively collected clinical data from JIA patients treated at the Children's Hospital of Chongqing Medical University between January 2013 and December 2022. To be included, patients should meet the following criteria: (i) the age of attendance is 14–18 years; (ii) the diagnosis of JIA for disease onset at <16 years of age met the 2001 International League for Rheumatology (ILAR) classification criteria (16) and for disease onset at ≥16 years of age met the 2018 Paediatric Rheumatology International Trials Organization (PRINTO) classification criteria (17). Exclusion criteria: (i) age at consultation <14 years old; (ii) lack of baseline data. According to ILAR criteria, JIA can be categorized into seven subtypes, including systemic JIA, rheumatoid factor (RF)-negative polyarthritis, RF-positive polyarthritis, oligoarthritis, psoriatic arthritis, enthesitis-related arthritis (ERA), and undifferentiated arthritis.

### 2.2 Data collection

The clinical data of the patients was retrospectively collected through our hospital's big data platform, including gender, age, disease duration, diagnoses, clinical manifestations, and laboratory indicators. Drug use includes non-steroidal anti-inflammatory drugs, glucocorticoids, conventional synthetic disease-modifying anti-rheumatic drugs (csDMARDs), biologic disease-modifying anti-rheumatic drugs (bDMARDs), Janus-activated kinase inhibitors (JAKi).

Disease activity was assessed by Wallace criteria (18). Inactive disease met the following conditions: (i) no joints with active arthritis; (ii) no fever, rash, serositis, splenomegaly, or generalized lymphadenopathy attributable to JIA; (iii) no active uveitis; (iv) normal ESR or CRP; (v) physician's global assessment of disease activity indicates no disease activity. Active arthritis was defined as a joint with swelling not due to bony enlargement or, if no swelling is present, limitation of motion accompanied by either pain on motion and/or tenderness. Clinical remission on medication was defined as inactive disease for a minimum of 6 continuous months on medication. Clinical remission off medicine was defined as inactive disease for a minimum of 12 continuous months while off all anti-arthritis and anti-uveitis medications.

### 2.3 Ethics

The Institutional Review Board of Children's Hospital of Chongqing Medical University approved the study [Approval No: (2023) IRB (STUDY) No.351]. The project was a retrospective study utilizing medical records obtained from previous clinical visits, without unnecessary risk to the patients.

### 2.4 Statistical analysis

Statistical analyses were performed using SPSS (Statistical Package for the Social Science; version 26). Continuous variables were described as the median value (IQR: 25th, 75th) and compared by the Mann–Whitney *U* test. Categorical variables, expressed as the number of cases and percentage (%), were compared by the chi-square test (*χ*^2^) or Fisher's exact test. *P*-values <0.05 were considered statistically significant.

## 3 Results

### 3.1 Clinical characteristics

We cumulatively collected 230 JIA patients from all over the country, including Chongqing, Sichuan, Guizhou, Yunnan, Fujian, Gansu, Henan, Shandong, Shanxi, and Xinjiang. One hundred and forty-four (63%) children were boys and eighty-six (37%) children were girls. The male-to-female ratio of the research was 1.7:1. For 2 cases of systemic JIA with onset after the age of 16 years old that met the PRINTO classification criteria were included in the systemic JIA group, and for 1 case of adhesion enthesitis/spondylitis-related JIA with onset after the age of 16 years old that met the PRINTO classification criteria were included in the ERA group. Thus, we ended up incorporating 73 (32%) ERA, 63 (27%) systemic JIA, 38 (17%) RF-negative polyarthritis, 33 (14%) RF-positive polyarthritis and 23 (10%) oligoarthritis. The mean age at the last follow-up was 15.71 years (14.77, 16.63), the mean age at onset was 11.86 years (9.05, 13.42), and the mean duration of disease was 4.06 years (2.07, 6.79).

### 3.2 Disease activity

**Figure 1:**
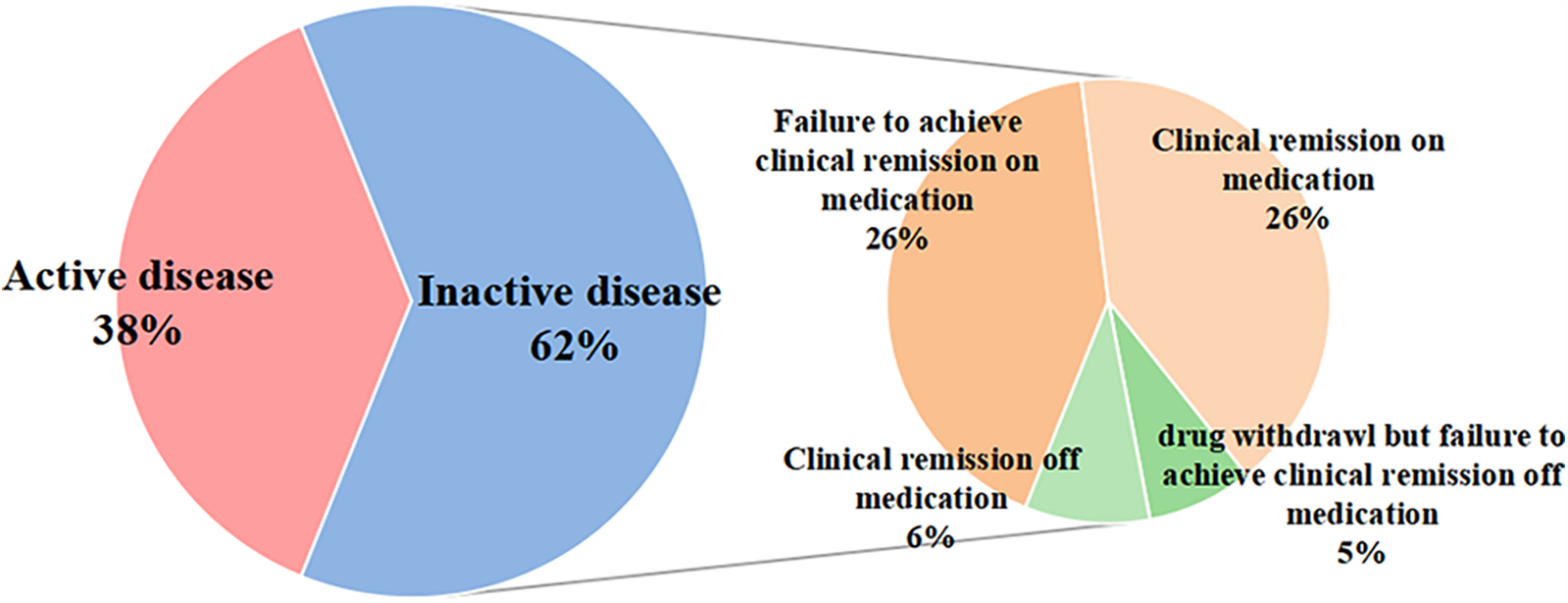
Disease activity during the pre-transfer period.

According to the medical record at the last pediatric visit, 65 (28%) patients showed signs of active arthritis, and only 1 patient with ERA had active uveitis. 87 (38%) patients with JIA were in disease active status and 143 (62%) patients were in disease inactive status (Figure 1). For patients with disease inactive status, 59 patients achieved clinical remission on medication, and 13 patients achieved clinical remission off medicine. In the present study, 44% of females were in active disease and 34% of males were in active disease (*P* = 0.124), and the difference was not statistically significant.

### 3.3 Treatment

**Figure 2:**
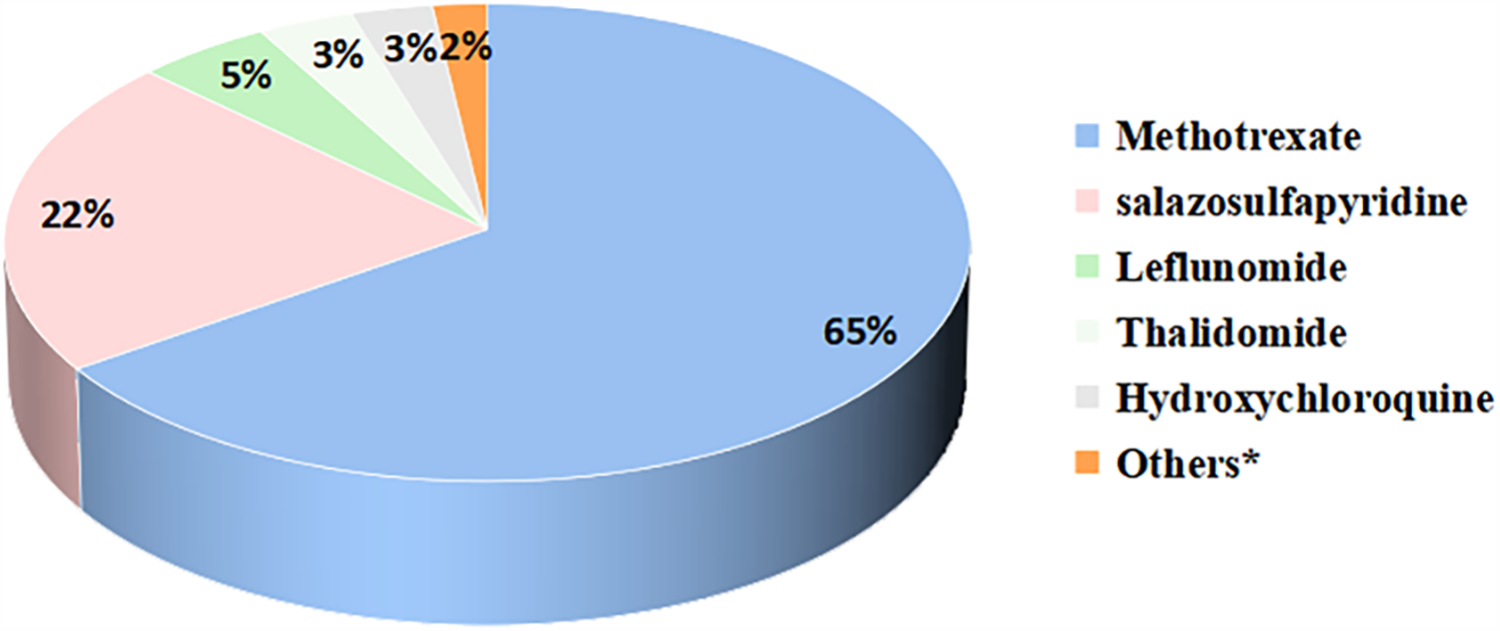
Proportion of csDMARDs applications during the pre-transfer period. *Others included colchicine, cyclosporine, cyclophosphamide, mycophenolate mofetil.

**Figure 3:**
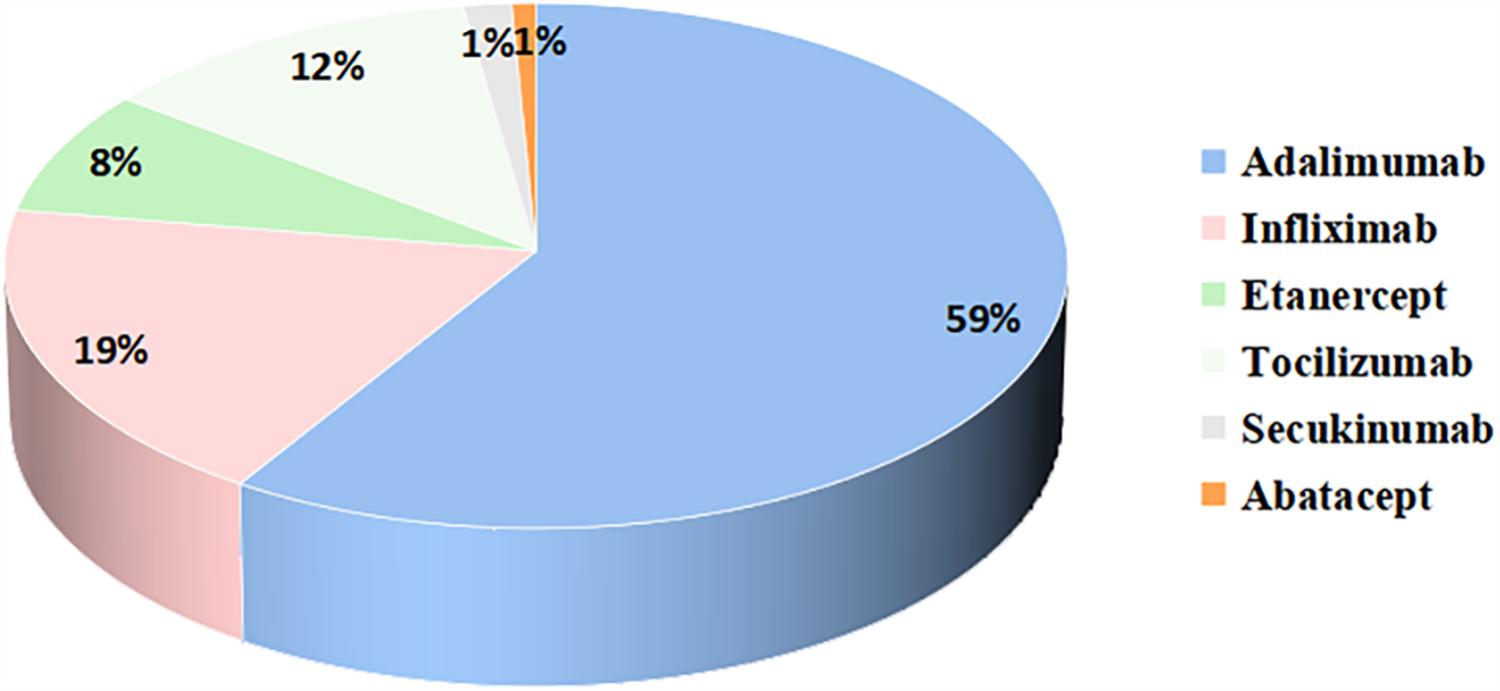
Proportion of biologics applications during the pre-transfer period.

Only 24 patients (10%) in our study achieved discontinuation of medication, with the majority of patients still requiring medication. 81 (35%) patients were still using non-steroidal anti-inflammatory drugs. Glucocorticoids were used in 28 (12%) patients. 191 (83%) patients were on csDMARDs. Methotrexate (65%) was the most commonly used csDMARDs, followed by salazosulfapyridine (22%), and other less commonly used csDMARDs included leflunomide, thalidomide, hydroxychloroquine, colchicine, cyclosporine, cyclophosphamide, and mycophenolate mofetil (Figure 2). 129 (56%) patients were on bDMARDs. Biologics included tumor necrosis factor inhibitors (TNFi), interleukin-6 receptor inhibitors, interleukin-17 receptor inhibitors, and abatacept (Figure 3). Adalimumab (59%) was the most frequently used biologics, followed by infliximab (19%), tocilizumab (13%) and etanercept (8%). 13 (6%) patients were on JAKi drugs, of which 10 patients were on tofacitinib and 3 were on baricitinib. A total of 123 patients were treated with both csDMARDs and bDMARDs, indicating that the majority of patients receiving biologics were also treated with csDMARDs.

A total of 51 patients switched biologic agents. Among them, 35 patients switched biologics once, 12 patients switched twice, and 4 patients switched three times. The most common switch was from one TNFi to another. The primary reason for switching biologic agents was treatment inefficacy (*n* = 49), while 2 patients switched biologics due to adverse reactions associated with Infliximab.

### 3.4 Comparison of different subgroups

The comparison of clinical features and therapy between different subgroups of JIA is shown in Table 1. Males outnumbered females in all subgroups except the polyarthritis group. The oligoarthritis group had an earlier onset (*P* = 0.020) and longer disease duration (*P* = 0.009) compared to other subtypes. The proportion of patients with active arthritis was significantly lower in the systemic JIA group (13%, *P* = 0.001) than in other subtypes. In contrast, RF-positive polyarthritis patients had a significantly lower rate of inactive disease (39%, *P* = 0.004) than the other subtypes and achieved a relatively lower proportion of clinical remission on medication or discontinuation of medication (18%, *P* = 0.024). The systemic JIA group had the highest discontinuation rate (22%, *P* = 0.000).

**Table 1:** Comparison of clinical features and therapy between different subgroups of JIA.

Variables	sJIA (*n* = 63)	oJIA (*n* = 23)	pJIA (RF−) (*n* = 38)	pJIA (RF+) (*n* = 33)	ERA (*n* = 73)
Male (%)	41 (65%)	12 (52%)	16 (42%)	8 (24%)	67 (92%)
Age of onset of disease (years)	11.07 (8.05, 13.96)	8.88 (7.39, 12.24)	11.59 (9.57, 12.85)	11.45 (9.12, 13.46)	12.71 (10.88, 13.85)
Age at follow-up (years)	16.05 (14.79, 16.87)	16.00 (14.57, 16.79)	15.56 (14.37, 16.47)	15.74 (14.69, 16.71)	15.52 (14.78, 16.40)
Duration of disease (years)	4.25 (2.27, 7.88)	6.40 (4.24, 8.67)	4.51 (2.21, 6.87)	4.29 (2.74, 6.32)	3.19 (1.80, 4.95)
Active arthritis (%)	8 (13%)	10 (43%)	11 (29%)	18 (55%)	18 (25%)
Disease Activity					
Active disease (%)	16 (25%)	11 (48%)	14 (37%)	20 (61%)	26 (36%)
Inactive disease (%)	47 (75%)	12 (52%)	24 (63%)	13 (39%)	47 (64%)
Clinical remission on medication (%)	13 (21%)	6 (26%)	11 (29%)	4 (12%)	25 (34%)
Discontinuation of medication (%)	14 (22%)	3 (13%)	3 (8%)	2 (6%)	2 (3%)
Clinical remission off medicine (%)	6 (10%)	3 (13%)	2 (5%)	1 (3%)	1 (1%)
Treatment					
NSAIDs (%)	16 (25%)	8 (35%)	12 (32%)	14 (42%)	31 (43%)
Glucocorticoids (%)	22 (35%)	1 (4%)	2 (5%)	3 (9%)	-
csDMARDs (%)	39 (62%)	18 (78%)	34 (90%)	31 (94%)	69 (95%)
One type (%)	30 (48%)	18 (78%)	29 (76%)	28 (85%)	69 (95%)
Two types (%)	9 (14%)	-	5 (13%)	3 (9%)	-
bDMARDs (%)	18 (29%)	12 (52%)	21 (55%)	25 (76%)	53 (73%)
IL-6 inhibitors (%)	13 (21%)	-	1 (3%)	2 (6%)	-
TNF inhibitors (%)	4 (6%)	12 (52%)	20 (53%)	23 (70%)	51 (70%)
JAK inhibitors (%)	7 (11%)	2 (9%)	1 (3%)	2 (6%)	1 (1%)

Data are median (IQR) and *n* (%) as appropriate. JIA, juvenile idiopathic arthritis; RF, rheumatoid factor; NSAIDs, non-steroidal anti-inflammatory drugs; csDMARDs, conventional synthetic disease-modifying anti-rheumatic drugs; bDMARDs, biologic disease-modifying anti-rheumatic drugs; IL-6, interleukin-6; TNF, tumor necrosis factor; JAK, Janus-activated kinase.

The proportion of csDMARDs (*P* = 0.000) and biologics (*P* = 0.000) utilization in the systemic JIA was lower than in the other subtypes. In the group of non-systemic JIA, the RF-positive polyarthritis group and ERA group had higher rates of csDMARDs (*P* = 0.048) and biologics (*P* = 0.010) utilization than the RF-negative polyarthritis and oligoarthritis groups. 17 (7%) patients were concomitantly using two kinds of csDMARDs, including 9 with systemic JIA, 5 with RF-negative polyarthritis, and 3 with RF-positive polyarthritis. Glucocorticoids, interleukin-6 inhibitors, and JAKi are commonly used in the systemic JIA group. Secukinumab was used only in the ERA group.

## 4 Discussion

This is the first study on the pre-transfer period of juvenile idiopathic arthritis in China, which is representative of southwest China. In this study, although the majority of JIA patients were in a state of inactive disease, only 6% of patients actually achieved clinical remission off medication. Therefore, it is critical for JIA adolescents to move from children's hospitals to adult rheumatology centers. Understanding the clinical characteristics and treatment of patients in the phase will facilitate a smooth transition for them.

Patients of JIA with different subtypes have different clinical manifestations. In previous studies, oligoarthritis was the most common subtype of JIA (19), whereas in our study oligoarthritis was less common. It was considered that patients with oligoarthritis had an early onset of the disease and a high rate of clinical remission after treatment. Comparing studies of JIA in adulthood, the adult JIA population is dominated by polyarthritis and ERA (1). In this study, systemic JIA, ERA, and polyarthritis each accounted for about one-third of the total, and in previous studies in Southeast Asia, systemic JIA and ERA were dominant (20). Studies from Taiwan, China, suggested that ERA is the predominant subtype (21). Epidemiologic data from the Chinese Mainland are still lacking, and our team is conducting relevant investigations and research. Considering that polyarthritis has a long treatment period and is difficult to discontinue taking medicine (22), the subtype distribution of JIA in our study was considered to be mainly related to age, geographic region, and prognosis of different subtypes.

As we all know, JIA affects more commonly girls than boys (ratio 2:1) (23). Whereas our study showed a higher percentage of JIA in boys than in girls (ratio 1.7:1), which was considered to be related to the distribution of JIA subtypes in this period. ERA often occurs in men, but that doesn't seem to be enough to fully explain such a big difference. The present study had higher rates of boys in each subtype than previous studies (24, 25), except for RF-positive polyarthritis. Adolescents in the pre-transfer period are also in puberty and have altered levels of sex hormones. Sex hormones affect both innate and adaptive immunity (26). Estrogens have both pro-inflammatory and anti-inflammatory effects (27). It remains unclear to what extent sex hormones influence the gender differences in JIA patients during this period.

Different subtypes of JIA have different ages of onset. Oligoarthritis is characterized by one of the clinical features of early onset, with a peak age of onset at 2–4 years (28). In our study, oligoarthritis had the earliest age of onset, but the median age of onset was 8.88 years. And there were only 12 cases (5%) of JIA starting at ≤6 years of age, and more JIA starting in late childhood or adolescence (mean age at onset was 11.86 years). This finding may indeed relate to our inclusion criteria, which required participants to be aged 14–18 years at their final follow-up visit. Such criteria inherently favor patients with later diagnoses. Importantly, our observations align with prior JIA cohort studies. Data from transition cohorts in Finland and Canada both showed that the age of onset of JIA was approximately 11 years (29, 30). As recommended by the guidelines, for patients diagnosed after the age of 14, preparation for transition to adult care should commence at the time of initial diagnosis (31).

In our study cohort, only 1 ERA patient had active uveitis at the last pediatric visit, which may be related to the timely application of biologics in treatment. Besides, the prevalence of JIA-associated uveitis (JIA-U) is generally lower in Asia compared to North America and Europe according to previous studies (32, 33). Despite identifying only one active uveitis case, our study emphasizes the necessity for vigilant monitoring and follow-up in JIA-U patients, as inadequate transitional care may lead to severe outcomes like blindness (9).

Disease activity of patients is closely related to criteria for disease activity assessment and treatment. A study from Finland (29), which had a similar csDMARDs utilization rates as our study, but only 29% for biologics, had a higher rate of active arthritis (40%) than the present study. Another study from Finland (34), however, had close csDMARDs and bDMARDs utilization rates to this study, but using DAS-CRP remission criteria, up to 89% of patients were in remission. There is a lack of relevant studies confirming exactly which criteria for assessing disease activity are more appropriate for JIA patients transitioning to adulthood. And previous study have shown that both the RA measures (including the Disease Activity Scores) and the Juvenile Arthritis Disease Activity Scores version show sufficiently good ability to categorize according to disease inactivity, but the study was not able to assess the clinical significance of the differences (35). Besides, medical resources in different regions may also have an impact on the disease activity of patients. From the ReACCh-Out cohort (30), the rates of anti-rheumatic medication use were 51% and biologics use were 22%, both lower than our study; however, also using the Wallace assessment criteria, 73% of the patients were in inactive disease, the rate of clinical remission without medicine was 47%, both higher than our study. In contrast, a study from Thailand about the transition period of pediatric rheumatic disease, which included JIA, had a csDMARDs utilization rate of 80% and a biologics utilization rate of 11% only, and as many as 78% of patients are in an active disease state (11). Previous studies have found that female patients during this period have higher disease activity than male patients (34). Female sex has also previously been reported to be associated with lower quality of life, and higher disability at 1 year of treatment (24, 36). However, no correlation between gender and disease activity was found in our study.

Among the biologics applications, the rate of TNFi application is as high as 85%. TNFi is widely used in the treatment of both pediatric and adult patients with JIA, and its efficacy and safety have been verified (4, 37–39). In previous reports, tumor necrosis factor antagonist application was dominated by etanercept (4, 38). However, in the present study, adalimumab was used most frequently. Differences in the use of biologics may be related to the economic situation and level of medical care in different regions. Tocilizumab was primarily used in systemic JIA, which was consistent with guideline recommendations (40). The most common biologic switch was from one TNFi to another, which is consistent with findings reported in the literature (41, 42).

Differences in disease activity and treatment existed between different subtypes of JIA. Patients in the RF-positive polyarthritis group and ERA patients had higher rates of csDMARDs and biologic application than other subtypes. The disease activity was significantly higher in the RF-positive polyarthritis group than in the other subtypes. In a Portuguese study of adult patients with JIA, RF-positive polyarthritis reported the worst functional outcome (1). However, more studies have shown that ERA patients had higher disease activity, poorer quality of life, and worse function (2, 43, 44). In our study, the disease activity of ERA patients was lower than that of patients in the RF-positive polyarthritis group, which was considered to be associated with the difference in disease duration between the two groups.

There may be selection bias in this study because it was a retrospective study. The cases selected were children who came to the hospital, so patients with high disease activity may have been included. Secondly, this study assessed the disease activity of JIA patients by Wallace criteria, which is a one-sided assessment of the disease. It is expected that prospective large-sample studies can be conducted in the future, and Juvenile Arthritis Disease Activity Scores (JADAS), Disease Activity Scores (DAS), Children Health Assessment Questionnaire (CHAQ), Transition Readiness Assessment Questionnaire (TRAQ) (45–48) and other evaluation methods or questionnaires can be used to comprehensively evaluate disease activity, quality of life, and transition readiness of JIA patients. Lastly, as a children's hospital, we only provide clinical data for patients aged 14–18 years. We hope to collaborate with adult rheumatology clinics in the future to establish transition clinics, ensuring a smooth transfer of care and providing more information about before and after transfer for JIA patients.

Overall, the majority of JIA patients in the pre-transfer period failed to achieve clinical remission and discontinuation of medication and required continued treatment. Conventional synthetic disease-modifying anti-rheumatic drugs and biologics were widely used in the treatment of JIA patients during this period. RF-positive polyarthritis group were less likely to achieve clinical remission. Our study is the first survey on JIA patients before transfer to adult care in China, which truly reflects the disease activity, csDMARDs and biologics application of JIA during this period. It is hoped that this study could provide a reference for seamlessly transitioning JIA patients to adult, as well as for rheumatologists and parents.

## Data Availability

The original contributions presented in the study are included in the article, further inquiries can be directed to the corresponding author.
